# What Decides Your Athletic Career?—Reflection from Our Study of GP.Mur-Associated Sports Talents during the COVID-19 Pandemic Era

**DOI:** 10.3390/ijerph191912691

**Published:** 2022-10-04

**Authors:** Kate Hsu, Wei-Chin Tseng

**Affiliations:** 1 The Laboratory of Immunogenetics, Department of Medical Research, Mackay Memorial Hospital, Tamsui, New Taipei City 251020, Taiwan; 2 Institute of Biomedical Sciences, MacKay Medical College, New Taipei City 25245, Taiwan; 3 Department of Nursing, MacKay Junior College of Medicine, Nursing, and Management, New Taipei City 25245, Taiwan; 4 Department of Exercise & Health Sciences, University of Taipei, Taipei 100234, Taiwan; 5 Department of Physical Education, University of Taipei, Taipei 111036, Taiwan

**Keywords:** psychological trait, environment, community, sport talent, sport gene, imagery, blood type, GP.Mur, career

## Abstract

This opinion article discusses the factors that attract children and teens to athletic careers. The most important attribute for the making of athletes is polished sports talent, followed by psychological, environmental, and incentive factors. Our laboratory studies a red blood cell (RBC) type called GP.Mur, which is rare in most parts of the world besides Southeast Asia. Intriguingly, the prevalence of the GP.Mur blood type is relatively high among Taiwanese elite athletes. The highest frequency of the GP.Mur blood type worldwide is found among Taiwan’s Ami people (88–95% from hospital blood bank surveys in the 1980s). Though the Ami constitute only 0.6–0.8% of the Taiwanese population, from records of national track-and-field games in the past century, 10–60% of the medalists were Ami. Biologically, GP.Mur expression supports blood CO_2_ metabolism, which may have implications for athleticism. As many of our study subjects are elite college athletes with the GP.Mur blood type, we contemplated their upbringings and career dilemmas, especially during the difficult COVID-19 pandemic. Beyond individual sports talent, the pandemic particularly tests personal characteristics and socioeconomic support for becoming an athlete.

## 1. Introduction

The choice of one’s career path is a complex issue, which is more difficult for gifted elite athletes who began training and competition at an early age. In this opinion article, we will discuss the making of an athlete in Taiwan from physical/genetic attributes (e.g., sport talent/gene) and psychological/environmental/incentive attributes, with emphases on imagery and family influence/tradition.

We study the biology and physiology of the GP.Mur blood type in Taiwan. GP.Mur is a unique Southeast Asian phenotype (rare outside Southeast Asia), with 2–6% prevalence in different regions of Taiwan [1,2], but with significantly higher prevalence in several tribes and on some elite sport teams (e.g., track, baseball, and soccer). Intriguingly, the biological function of GP.Mur is to promote blood CO_2_ respiration, which conceivably benefits exercise metabolism. In Taiwan, junior athletes who have won competitions at national and international games are granted scholarships and are invited to continue their training at top sports universities. Like piano prodigies, their athletic talents are often revealed at a young age. Different from piano prodigies though, their athletic careers are limited by age (usually through their early 20s) and injury. When elite athletes no longer meet the cutoff for national/international games and/or have other considerations, they have to prepare for a career change to other sport-related or unrelated areas, often while they are still in college. For the making of one’s athletic career, we will discuss (1) the attributes of potential sport talents, using two examples: the well-studied *ACTN* polymorphism and the commonly observed *GYPB/GYP.Mur* polymorphism in Taiwan; and (2) the attributes of one’s mentality and environment. We will also discuss the interplay among these attributes in the contexts of the COVID-19 pandemic and GP.Mur-associated athleticism in Taiwan.

## 2. Impacts of COVID-19 Pandemic on Taiwanese Sports and Athletes

Since February, 2020, the COVID-19 pandemic and related public health measures have forced the reduction of sporting events, tournaments, and even gym/training classes in most parts of the world [3]. This has taken away chances of competitions that many athletes have long prepared for, devastating their livelihoods [4,5,6,7]. However, in Taiwan, COVID-19 had rather minimal influences among Taiwanese athletes or the daily lives of Taiwanese before the spring of 2022 (<0.1% of the population was infected with COVID-19 during December 2019–March 2022). This is due to a prior experience with the devastating SARS coronavirus in 2003 that resulted in a mortality rate of 27% in Taiwan [8]. When facing a similar coronavirus in December 2019, the Taiwanese government acted quickly to impose a mask mandate and border control at airports and harbors, to halt the entry of the COVID-19 virus to Taiwan’s islands [9]. Omicron variants, however, were unstoppable at the borders, despite these public health measures. By the end of the summer of 2022, ~24% of Taiwan’s residents had been infected with COVID-19, and ~0.0043% had lost their lives from COVID-19 or related complications [10].

In 2021 (the second year of the COVID-19 pandemic), most international games were either resumed after a postponement (e.g., the 2020 Tokyo Olympic were postponed to 2021) or were unaffected. The Taiwan Centers for Disease Control (TW-CDC) maintained its rigid public health measures, and all international games that were planned to be held in Taiwan from 2020 to the spring of 2022 were canceled. During the same period (late 2020–early 2022), the Taiwan Sports Administration, under the advice of the TW-CDC, discouraged elite athletes from going overseas for competitions held in other countries. Many elite athletes needed to fight against this government policy by promising to take any risks themselves, in order to participate in overseas tournaments. At the 2021 Asian Karate Federation (AKF) championship tournaments in Kazakhstan, more than half of the Taiwan karate team members unfortunately contracted Omicron after the competition, despite the fact that all the team members had been vaccinated [11]. Like elite athletes in other countries, their COVID-19 infections were generally mild (i.e., fatigue as the main complaint) [12]. Although their symptoms did not seem as severe as nonathletes of the same age, it is unclear how this respiratory virus could affect their cardiopulmonary functions in the long run. Currently, their exercise capacity is estimated by lactate-based calculation for anaerobic thresholds (AT) and not by cardiopulmonary exercise testing (CPET) that measures VO_2_max directly. This is because CPET face masks and gas tubes are shared equipment, so any residual infectious particles on the shared equipment may not be eliminated by standard cleaning protocols.

The restrictions on international games were lifted as Taiwan relaxed its border control in the summer of 2022. The YONEX Taipei Open 2022 (a Badminton World Federation (BWF) world tournament) was the first international games to be held in Taiwan since the beginning of the pandemic. Currently, the precautionary measures against COVID-19′s spread via sport games are quite different (i.e., whether rapid testing should be required before a game; whether “sport bubbles” should be imposed on foreign participants); the public health regulations depend on the type of sport and on the national policy of the country where the games are held. 

The pandemic impacts individual Taiwanese athletes to different extents. In the beginning of the outbreak (the spring of 2020), schools were closed abruptly and all sports training and coaching classes were halted for the summer. The Taiwan National Intercollegiate Athletic Games of 2020 were postponed from spring to winter. Noticeably, at the postponed intercollegiate games, student-athletes who trained by themselves or through online coaching generally did not perform well. The situation is similar to a recent report describing the plummet of math and reading scores among elementary school children during COVID-19 lockdowns in the USA [13]. Students need in-person guidance and discipline. Though student-athletes have always been trained and motivated to win, they are also “lazy” by nature like nonathlete students [14], according to a local coach. 

On the other hand, the performance of Taiwanese elite athletes (including those of college age) who need to accumulate world ranking scores has not been much affected by the lockdowns. Conceivably, their psychological strength, from becoming world-ranking athletes (e.g., mental stability and grit [15]), manifested itself particularly during the difficult and uncertain pandemic era. Some world-ranking athletes have also been personally paying their coaches and their own tournament/travel expenses. For them, their athletic accomplishments are more than careers that earn their livings. Besides, unlike other professionals, the duration of one’s athletic career is limited by the age of prime performance, never mind that the bar for becoming an elite or professional athlete is very high [16]. Athletes who wished to remain competitive during the pandemic era needed to keep their peak performance and be more self-motivated than ever. Even before COVID-19, elite athletes who were serious about their sporting careers had been trying to extend the duration of their peak performance beyond the optimal age for competition [16].

## 3. Polished Sport Talent as the Most Important Attribute in the Making of Elite Athletes

The most essential attribute to become an elite athlete is one’s polished sports talent, with a suitable physical build for the type of sport competition that one aims for. Such qualities are, by and large, genetically determined. We will discuss two gene polymorphisms related to potential sport talents: (1) *ACTN* and (2) *GYPB/GYP.Mur* (the latter gene variant encoding GP.Mur). However, it would be a stretch to describe these variant genes as “sport genes”.

The RR/RX/XX polymorphism of *ACTN3* alleles was initially found to be associated with differential muscle utilities for sprinting versus endurance sports: the RR/RX genotypes likely benefit speed and power athletes, whereas the XX genotype likely benefits endurance athletes [17,18,19,20]. People with *ACTN3* RR/RX genotypes express alpha-actinin-3 in the Z disc of their myofibers, whereas people with the homozygous *ACTN3* R577X (XX) genotype do not express alpha-actinin-3 at all. The absence of alpha-actinin-3 in humans is not detrimental to health. In fact, 25% of Asians, 18% of Caucasians, and 3% of African Americans carry the XX genotype and lack alpha-actinin-3 protein in their myofibers. 

However, further *ACTN3* molecular studies using genetically “knocked-out” mouse models revealed a more complex picture than researchers initially thought. Knock-out, or removal of *ACTN3* gene expression, reduces the fraction of fast myofibers in total muscles and lowers the total muscle mass of genetically modified animals. Not surprisingly, *ACTN3^−/−^* mice show weaker skeletal muscle strength than wild-type mice [21]. In a more recent mouse study using different molecular biology approaches, *ACTN3* gene doping does not enhance muscle function, instead it induces toxicity that is harmful to the gene-doped muscles [22]. There are also controversial reports from human studies in various athletic cohorts worldwide, which did not find a significant correlation between *ACTN3* genotypes and sport types [17,18,23,24,25,26]. As David Epstein quoted from Carl Foster of the Human Performance Laboratory at the University of Wisconsin: perhaps the best genetic test for sports talents is still using a stopwatch [27]. The concept of “sports genes” alone could not fully explain the complexity of human (or rodent) exercise behavior.

**Figure 1:**
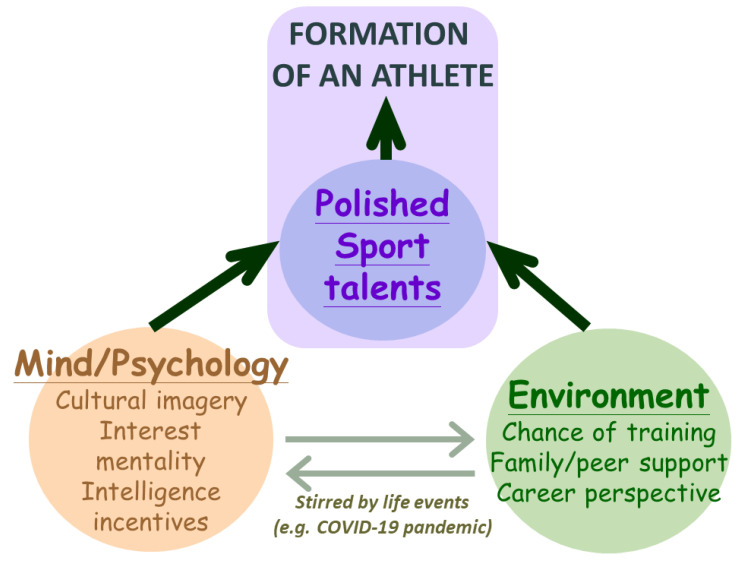
The most important attribute for the formation of an athlete is one’s polished sports talent, which is supported or discouraged by environmental and psychological factors. One’s environment and mentality can be influenced dynamically by life or world events, such as the COVID-19 pandemic.

Besides individual sports talents, there are other essential attributes for the making of elite athletes: (1) an environment that helps nurture one’s sports talents; and (2) one’s mentality, which is largely driven by personal interests, psychological traits, willpower/motivation [28], intelligence [29], and realistic incentives. The interplay between these environmental and psychological attributes influences how an athlete responds to the hardships of training and setbacks and still decides to courageously stay for competition (Figure 1). During the quarantine and lockdown of the pandemic, athletes had to be mentally stronger and even more creative to keep up [29,30,31]. In the long run, however, accumulated mental and socioeconomic stress could be detrimental to maintaining one’s peak performance or even to keeping one’s athletic career [4,5,31]. The pandemic is likened to an invisible test that further select athletes by their mental capacity (i.e., resilience, and intelligence/creativity), as well as socioeconomic and emotional support from their families, their communities, and society (Figure 1).

## 4. GP.Mur Blood Type as a Potential Inborn Physical Advantage

Similar to *ACTN3* mentioned above, *GYP.Mur*-encoded GP.Mur has been suggested as a potentially characteristic phenotype for athleticism in Taiwan. GP.Mur, traditionally known as Miltenberger subtype III (Mi.III), is the protein entity of a special blood type that is found in 1–7% of Southeast Asians but is considered rare in other ethnic populations (e.g., <0.01% in Caucasians and Northern Asians (Japanese, Korean, and northern Chinese)) [1,32,33]. Southeast Asian blood banks pay special attention to it, because RBC antigen incompatibility involving GP.Mur could occur during a transfusion or during a second or later pregnancy and trigger acute intravascular hemolytic reaction [32,34,35,36,37]. The highest frequency of the GP.Mur blood type worldwide is found in Taiwan’s Ami people (88–95% from hospital blood bank surveys in 1980s) [38]. The Ami is a plain tribe that, nowadays, is scattered in various places throughout Taiwan and constitutes about 0.6–0.8% of the Taiwanese population. 

However, the regional and worldwide distribution of the GP.Mur phenotype is changing. The Australian Red Cross Blood Service recently did a survey on a blood donor population and found 0.22% of the donors express the Mi^a^ antigen (a main marker of the GP.Mur blood type) [39]. This reported frequency in Australia is ~20 times higher than Western surveys done before the 1990s [33] and is likely due to increasing immigration from Southeast Asia and international marriages. From the perspective of population genetics, the concentration of a genotype in a historically isolated population (e.g., *GYP.Mur* in the Ami people) could be a consequence of the founder effect. However, this phenomenon is affected by increasing human migratory activities (e.g., urbanization and globalization), as demonstrated in Australia [33,39] and in the changing regional distribution of GP.Mur prevalence in Taiwan [2,38]. 

**Figure 2:**
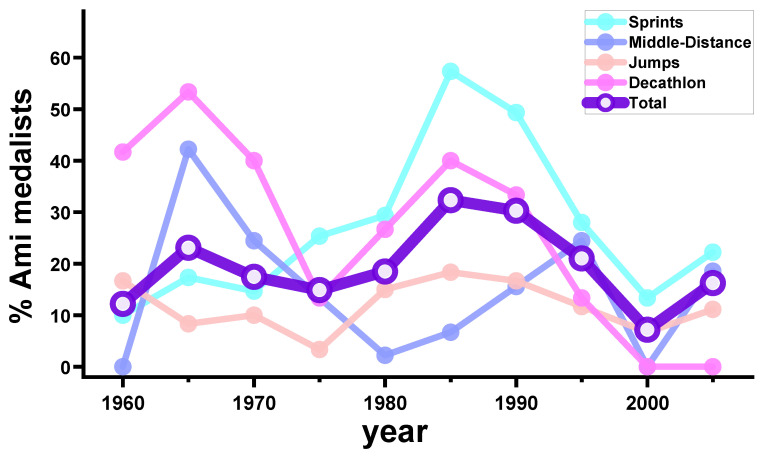
The percentages of Ami medalists (top-three winners) in Taiwanese national track-and-field games from 1957 to 2005 are disproportional to the mere 0.6–0.8% population of Ami in Taiwan [41]. Each datum point is the percentage of Ami male medalists among all male winners in a 5-year timeframe (i.e., the datum point at 2000 represents the number from 5-year compilation from 1996 to 2000). The “Sprints” section includes 5 types of games—100 m, 200 m, 400 m, 100 m hurdles, and 400 m hurdles. For each datum point in the “Sprints” section, the denominator is 75 (3 medalists/type × 5 types × 5 years). The same rationale applied for the calculation of other sections specified in the graph. The “Middle-Distance” section includes 800 m, 1500 m, and 3000 m steeplechase. The “Jumps” section includes long jump, triple jump, high jump, and pole vault. The “Total” encompasses statistics from the four sections—Sprints, Middle-Distance, Jumps, and Decathlon.

From the perspectives of physical build and strength, Ami people are not distinct from other Taiwanese in terms of muscular or anthropometric characteristics [40]. Yet, from records of Taiwan national track-and-field games in the past century, 10–60% of the medal winners were Ami (Figure 2) [41]. The percentages of Ami medalists were up to 50–60% in decathlon and sprinting events (Figure 2). A special report once described Ami decathletes, basketball players, and baseball players to be gifted with mystically unceasing energy and abnormally fast recovery after prolonged competition [42]. The decathlon legend C. K. Yang, nicknamed “the iron man of Asia” on the cover of *Sports Illustrated* in the 1960s [43,44], was Ami. Intriguingly, a century ago, the Japanese colonial government in Taiwan (1895–1945) had already documented the physical superiority of the Ami in forced hard labor, baseball, and physical education classes [41,45,46,47,48]. The imagery between the Ami people and athleticism existed long before our GP.Mur research suggested a possible biological link.

In 2009, in collaboration with a renowned proteomics research group in the USA, we identified significantly more band 3 protein expressed on the surface of GP.Mur RBCs, using an at-the-time novel system biology approach [49]. Band 3 is a Cl^−^/HCO_3_^−^ transporter, a functional membrane protein that allows the HCO_3_^−^ anion to pass through the cell membrane (ions or charged molecules cannot pass through a lipid membrane because of a tremendous thermodynamic energy barrier). Physiologically, HCO_3_^−^ transport via band 3 supports blood CO_2_ metabolism and respiration [49], because up to 90% CO_2_ in human circulation is present in the form of soluble HCO_3_^−^ before CO_2(g)_ is exhaled from the lungs. More band 3 expressed on the GP.Mur RBC surface means more efficient HCO_3_^−^ transport in and out of the RBCs and expedited blood CO_2_ metabolism.

The clinical relevance of band 3 was first discovered in cases of hereditary band 3 deficiency or dysfunction (e.g., Southeast Asian Ovalocytosis [SAO]; rare RBC En(a-) and M^k^M^k^ types); all these illnesses present lower blood CO_2_ respiration, mild acidosis, and, consequently, anemia due to low band 3 function in HCO_3_^−^ transport [50,51,52,53,54]. A complete lack of band 3 is naturally lethal for humans, unless aggressive medical interventions, i.e., in utero transfusion and postnatal routine transfusion, are implemented [55,56]. In contrast, in people with the GP.Mur blood type, their RBC membrane expresses more functional band 3 and, hence, a larger capacity of Cl^−^/HCO_3_^−^ exchange, superior intracellular pH homeostasis, and more resilient RBC membrane structure to osmotic stress [49,57,58]. The long-standing imagery between the Ami people (88–95% GP.Mur) and athleticism prompted us to examine the potential contribution of the GP.Mur phenotype to its carriers’ physical advantages. We tested nonathlete GP.Mur carriers and control subjects using a standard 3 min stepping test (a version of the Forestry Step Test). The subjects with the GP.Mur blood type (despite being Ami or not) showed faster CO_2_ respiration than those without the special blood type in this test [59]. Interestingly, from interaction and conversation with the test subjects, some participants with the GP.Mur blood type reflected that they generally do not become tired easily from strenuous physical activities compared to their friends, reminiscent of the observation documented by colonial Japanese officials over a century ago [41,45,46,47,48]. Conceivably, faster CO_2_ respiration in these subjects with GP.Mur could enable them to recover from physical stress faster than the control subjects.

## 5. Psychological and Socioeconomic Attributes in the Formation of Athletes

The human physiology study on GP.Mur [59] was reported by the Taiwanese media during the 2017 Summer Universiade Games—Taipei. *Is there indeed a correlation between the physiological advantage associated with the GP.Mur blood type and the making of an elite athlete?* Though the historical record (Figure 2) and our experimental finding implicate a potential correlation, athletic performance is much more than sport gene expression. A better question perhaps is: *What is the weight of sports talent in the making of an athlete?*

In 2012, the National Taiwan Sport University (NTSU) surveyed the distribution of the GP.Mur blood type on several athletic teams. NTSU is one of the top three athlete-training schools in Taiwan that recruits the best student-athletes from the winner pools of international, national, and regional games. Their survey did not find any GP.Mur+ athletes on the golf team or the jump team in NTSU. Among the 28 sprinters that specialized in short-distance hurdles, 9 of them (32%) bore the GP.Mur blood type, and 2 of the 9 GP.Mur+ sprinters were not Ami (11%, which was 2–3 folds higher than in the general population). The prevalence of the GP.Mur phenotype was drastically different on different sports teams. At another top-ranking sport university in Taiwan—the University of Taipei—there are elite athletes with the GP.Mur blood type that specialize in jumping, javelin, karate, judo, soccer, basketball, soft tennis, triathlon, cycling, canoeing, and weight-lifting. However, we have not yet found an elite athlete with GP.Mur on the golf, archery, or swimming teams. The latter was a surprise, as our biochemical work points to the potential benefits of GP.Mur protein expression for respiration [49]. From communication with the coaches/trainers of the swimming teams at the University of Taipei and the Chinese Culture University, elite swimmers are trained to endure hypercapnia, or high CO_2_. How GP.Mur supports exercise respiration mechanistically is perhaps different from what we initially envisioned.

The other two sports that lack GP.Mur+ athletes are gold and archery; most Taiwanese do not learn either sport before college. Golf is a more popular but also a more costly sport. Thus, choosing a specialty for an athlete also depends on the environment that one grew up in, e.g., the expertise of their first sports coach at the elementary or junior high school, and the influences and imagery from their families, their communities, and online media.

A 2012 NTSU survey found 28% of their baseball team members bore GP.Mur (7/25), which is ~6–7 times higher than the prevalence of GP.Mur in the general Taiwanese population. All seven of these GP.Mur+ baseball players are Ami, but there are two other Ami on the baseball team who do not have the GP.Mur blood type. The Ami’s baseball talents were first uncovered by Japanese ruling officials in Taiwan over a century ago (as seen in the movie *Kano* [60]). Nowadays, the exceedingly high percentage of Ami elite baseball players could also be driven by cultural influences, such as the imagery with family/tribal/community heroes, neighbors, and peers, and a fostering environment for baseball at the elementary school (Figure 1). For the making of elite baseball players in Taiwan, these environmental and cultural attributes may outweigh an inborn physical advantage such as GP.Mur.

One notable distinction between baseball players and sprinters in Taiwan is their career paths, which are strongly influenced by individual athletic talents, personality, and realistic incentives. In contrast to the fun of ball games and the relatively longer career span of a baseball player, the competing years of a sprinter usually end before their college graduation [16]. It is common for top Taiwanese track-and-field athletes who wish to remain in competition to extend their college years or postpone graduation in order to be continually supported with scholarships and professional training provided by the school, the government, and sponsoring companies. Seasoned college athletes need to weigh these incentives (training resources, coverage of expenditure for competition, sponsorship/paycheck, and future career aspects), along with life goals, family responsibility and other aspects.

The professional baseball league in Taiwan has been booming for more than two decades. Taiwan’s professional baseball league was also the first in the world to be out of COVID-19 lockdown, resuming play in an “empty stadium” in April 2020 [61]. Though they are primarily for broadcasting revenues and visibility, live baseball games provide a public emotional outlet. From this perspective, baseball players have an important social function, and this career is favored particularly as student-athletes grow older. 

Athletes in group games such as baseball, basketball, and even boxing are generally more receptive to cheers and emotional responses from the audience and teammates. This is in contrast to athletes trained for solo competition such as triathlons, marathons, and even swimming, who are more used to practicing in solitude. Thus, one’s personality is perhaps the second-most-important determinant, following inborn sport talents, for choosing one’s career path. The challenges of COVID-19 lockdowns accentuated the importance of certain personality traits, since the practice of social distancing is psychologically stressful particularly for some athletes. As with other careers, athletes may also “work (train) from home” to avoid infection. Athletic training in solitude is, thus, a test of persistency and motivation (psychological strength) and of flexibility or even independent problem-solving skills (creativity/intelligence) [6,7,29]. Overall, athletes that are superior in creativity, intelligence, grit, and mental stability, as well as in financial and family support, may perform relatively better during a crisis or difficult situation [15,62].

Sports performance is more than a manifestation of “sports gene” expression; it is a highly motivated and well-orchestrated human behavior. What drives a child with tremendous sports potential to become a runner or a baseball player is highly individualized. Though the sports talents in the Ami or GP.Mur+ people have been observed for more than a century, fewer of them are pursuing athletic careers nowadays, as shown in the decreasing percentages of Ami medalists since the 1990s (Figure 2). This dropping number could be explained by the rapidly changing society and culture. There are more diverse career paths and choices for youngsters to explore, though the biological advantage of GP.Mur in its carriers is still there. We once interviewed two equally talented sprint athletes before college, with career perspectives that became entirely different after they entered top sport universities. One was a GP.Mur+ double champion in short-distance and middle-distance run. With a goal of extending his running career to 40 years of age or beyond, he switched to training for long-distance run and marathons in college and continued to win medals in these new areas of competition. His determination and passion for running conceivably helped him develop versatile running talents. The reward from winning competitions was an important incentive; yet, injury from overtraining and/or over-competition was almost unavoidable. The other interviewee was a short-distance run champion that had made an Olympic training camp before college. Before his main competition, however, he decided to leave the Olympic camp, as he believed they had already achieved his best sprint performance in life. At the time of our interview, this ex-Olympic trainee has been planning for years to study alternative music overseas, after graduating from a top athlete-training university. The second interviewee also lacked the cultural imagery for a sport career that the first interviewee grew up with.

## 6. Conclusions

**Figure 3:**
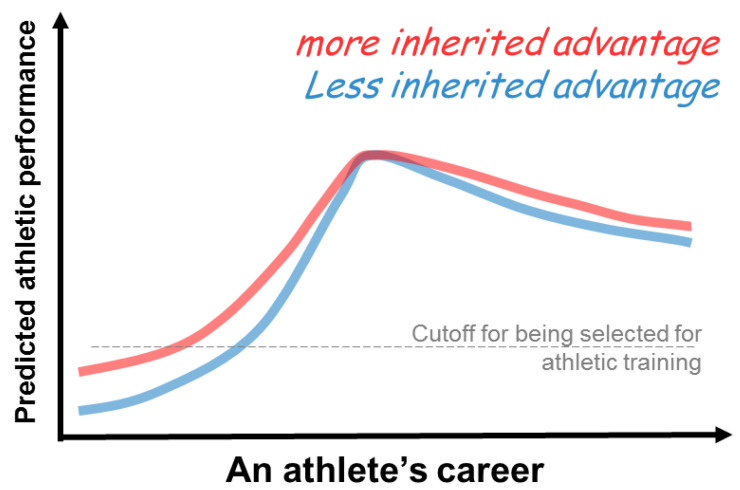
A hypothetical plot for athletic performance over an athlete’s career span. The footing for those that inherited more sports talent (red line) is deemed higher than the footing for those with less sports talent (blue line). If there is no external interference throughout, the difference between the red and blue curves is expected to be smaller at the peaks. Conceivably, one with less talent would need to put in more effort to reach one’s peak level, as demonstrated by the steeper upward slope of the blue curve compared to the slope of the red curve.

A child’s gift of inborn athletic advantages (“sports genes/talents”) conceivably could help the child to be selected into a sports team more easily (as shown in a hypothetical plot in Figure 3: an athlete that is more talented (shown as the red curve) begins with a higher footing). Regardless of having more or less athletic talent, one needs to put in serious training efforts in order to win a competition. Even though the amount of effort required to reach the same level of athletic abilities is deemed different between those with more sports talent and those with less, how long an elite athlete can stay competitive is often less affected by their inherited advantages (Figure 3: the difference between the blue and red curves hypothetically becomes smaller at the peak). Instead, the shape of an athlete’s performance curve and the duration of their athletic career are determined by individual psychological and environmental attributes, as well as realistic incentives (Figure 1). An example of how these factors are intertwined is the issue of pregnancy and maternity leave in elite female athletes, as demonstrated by Olympians Allyson Felix and Alysia Montaño from the USA [63]. Though gender-specific topics in athleticism are not covered in this article, it is a complex dilemma to weigh families and communities, paychecks and health insurance, and personal health and athletic performance, as well as chances for lifetime achievement, when deciding one’s career.

Therefore, one’s athletic career is highly individualized, often likened to running a “one-person company” with a diverse advisory board. How the company performs and how long it lasts depend on how the company is managed and developed. Besides one’s athletic performance, environmental and community supports are essential, but individual character and determination, which are shaped by personal beliefs and thoughts, are likely the ultimate attributes to deciding one’s athletic career. We noticed interesting tattoos on some of our athlete subjects, when they were tested on a treadmill for exercise cardiopulmonary functions. A waterweed-like tattoo on a professional soccer player once caught our attention, and later we learned that it represents a special grass from his hometown far away. His teammate has tattoos on both arms: on one arm is the symbol of a cross and on the other arm is an unknown English word, which was explained as being the name of his grandmother, whom he missed very much. These tattoo imageries are endearingly personal and almost spiritual. Despite that retirement or career change is unavoidable for athletes, the sports talent that once flourished in competition never really perishes; it has just been transformed as one pursues new directions in life. A friend who taught artistically gifted children once said that talented kids are not gifted in just one area but are often gifted in many areas. Indeed, the development of one’s athletic talent helps cultivate other talents and characteristics, such as grit [62]. In spite of numerous life challenges, such as the current COVID-19 pandemic, the ultimate factors in deciding one’s athletic career and beyond are, perhaps, rooted primarily in personal beliefs and characteristics that were co-developed with one’s athletic talent in the early years [15,62,64].

## Data Availability

Not applicable.
